# Book review of artificial intelligence in medicine Edited by Lei Xing, Maryellen Giger, and James Min

**DOI:** 10.1002/acm2.13157

**Published:** 2021-01-05

**Authors:** Dandan Zheng

## 1 OVERVIEW

The past decade has been a golden age for artificial intelligence (AI), with an explosive amount of research and development. From AlphaGo to self‐driving vehicles and brain–computer interface, transformative changes and bold ideas are sweeping through our everyday lives. One important application area is the field of medicine. In medicine, AI has also dominated scientific conferences in recent years, especially for medical physics as medical physicists have always been technological frontrunners among medical professionals. However, in contrast to the mounting literature on medical AI research, there have been few books systematically addressing the topic. In 2020, Elsevier published a timely new book, *Artificial Intelligence in Medicine*, successfully filling this gap. The book is an epic volume for professional and scientific readers interested in the topic.

This excellent book was edited by three international champions of the field, Dr. Lei Xing of Stanford University, Dr. Maryellen Giger of University of Chicago, and Dr. James Min of Cleerly, Inc. The chapters were contributed by a few dozen known experts in the field, such as Dr. Bradley Erickson, Dr. Ge Wang, Dr. Philippe Lambin, and Dr. Lily Peng. This comprehensive and authoritative book provides a great foundation for anyone interested in researching, developing, or using medical AI technologies.

## 2 PURPOSE AND AUDIENCE

The purpose of the book is to provide a comprehensive overview of the fundamental principles, technical basis, clinical applications, and practical considerations of AI in medicine. The expert author team takes a multidisciplinary approach, addressing a wide range of AI applications in medicine, such as preventive medicine, disease management, monitoring of patient status, imaging, biomarker discovery, drug design and repurposing, healthy living, elderly care, robotic interventions, and AI‐augmented telemedicine. While lay audience may also find the book informative and useful, the main target readers of this book are professionals in the field — students, researchers, engineers, clinicians, etc. With the panoramic overview and in‐depth discussions provided by the book, readers could attain useful background knowledge, learn about emerging computing algorithms, gain practical perspectives, and appreciate the current challenges and opportunities of AI in medicine.

## 3 CONTENTS AND HIGHLIGHTS

This book is 544 pages and contains a total of 25 chapters divided into the following four parts: Introduction, Technical Basis, Clinical Applications, and Future Outlook. The book begins with two introductory chapters, one accounting the history of healthcare AI and the future it leads to, and the other briefly laying out the key methods and tools as well as the clinical applications, drawing a distinction between the deep machine learning methods powering the AI developments emphasized in this book and the previous rule‐based and probabilistic methods. The Technical Basis part contains five chapters, discussing the fundamentals of video data‐based deep learning, imaging data‐based deep learning, medical expert systems, distributed learning, and analytics for multimodal data integration. These first three chapters build a helpful technical foundation for the clinical applications to be covered in the next part, and the following chapters introduce two important topics and directions — (a) distributed learning for multi‐institutional training to overcome the critical problem of data size limitation and the privacy hurdle in patient data sharing, and (b) multimodal data integration analytics for effectively integrating the multiomics and clinical data to maximize the synergy of big‐data biology and medicine. The next part, Clinical Applications, is the core of the book, spanning over 300 pages and containing 15 chapters. A wide range of topics are covered in this part, ranging from the imaging‐based applications that have pioneered the success of medical AI — those in breast cancer, radiology, pathology, GI endoscopy, retinal fundus photography for diabetic retinopathy detection, cardiovascular systems, and personalized and precision cancer diagnosis and management, to other wider ranging topics such as electronic health record data mining, wellness sensing, smart phone applications, public health surveillance, urology, oncology, pediatrics, and clinical neurological conditions. Each of these chapters details the state‐of‐the‐art developments within the application area, and also discusses the authors’ outlook of the challenges and the future. It is interesting to see how AI agents and their success vary among different fields due to the variabilities in data availability, data format, task, and end goal. The last part of the book consists of three chapters, providing focused discussions on regulatory, social, ethical, and legal issues of medical AI, industry perspectives and commercial opportunities, and future outlook and challenges. Social and regulatory considerations are important for adoption and trust in medical AI, so the first concluding chapter details these factors and promotes a new regulatory model that focuses on regulating the process rather than the product. The second concluding chapter switches perspectives from the scientific focus of the book to industry perspectives, introducing the “AI booms and busts cycle” for development and innovation, reviewing current capital investments and business opportunities, and discussing future trends of AI. In the final chapter, the editors once again overview the wide‐ranging applications addressed in the book, and discuss the challenges and future directions of AI‐powered medicine. On this outlook, the future generation of medical AI is expected to better converge with human intelligence and be more generalizable, interpretable, transparent, and trustworthy.

## 4 CRITICAL ASSESSMENT

Packed full of latest developments and expert discussions, *Artificial Intelligence in Medicine* covers a wide range of general and specialty medical AI topics. The discussed medical AI applications are fueled with a vast range of medical big data such as imaging, video, text, audio, genomics, demographics, and laboratory measurements, and these include both standard medical data and those mass collected from mobile devices. In addition to covering the technical basis and computing algorithms, the book also details the unique success and challenges in each clinical area ranging from specific medical problems, different health specialties, to global epidemic monitoring and control. The book also folds in the regulatory and industry perspectives as well as experts’ outlook of the future of medical AI.

Compared with similar books on the market, *Artificial Intelligence in Medicine* is uniquely suited as a foundation reading for anyone interested in working with AI in medicine. Unlike single‐author books such as *Deep Medicine, Artificial Intelligence in Healthcare, and Machine Learning and AI for Healthcare* that were written to introduce the medical AI topic to and engage a general discussion with lay audience, this book is a comprehensive, scientific, and technical volume contributed by a large team of expert authors for professional and scientific audience. Compared with other relevant technical books such as *Big Data in Radiation Oncology, Radiomics and Radiogenomics, and Machine Learning in Radiation Oncology*, this book goes beyond radiation oncology and medical physics to comprehensively cover the wide field of medicine. For medical physics readers, the book not only touches on the applications familiar to us but also offers valuable inspirations from those others that we may not usually encounter in our specialty. The wide range of applications and topics addressed by the book could help readers from any field to frame a global perspective on AI in medicine.

The field of AI is rapidly evolving, making any paper publication difficult to encompass this ever‐changing body of knowledge. Released in September 2020, *Artificial Intelligence in Medicine* provides the best available comprehensive and fundamental volume on the topic. The book highlights a current dichotomy: despite the enormous promise AI holds in medicine, it has yet to show revolutionary clinical benefits, indicating that we may still be at the dawn of a new AI age in medicine. Written by a large team of influential editors and authors, *Artificial Intelligence in Medicine* undoubtedly provides a great resource for anyone interested in playing a part in this exciting, upcoming new age.

